# Synthesis of Key Fragments of Amphidinolide Q — A Cytotoxic 12-membered Macrolide

**DOI:** 10.3390/molecules16075422

**Published:** 2011-06-27

**Authors:** Kohei Kawa, Akihiro Hara, Yuichi Ishikawa, Shigeru Nishiyama

**Affiliations:** Department of Chemistry, Faculty of Science and Technology, Keio University, Hiyoshi 3-14-1, Kohoku-ku, Yokohama 223-8522, Japan

**Keywords:** amphidinolide Q, *Amphidinium* sp., macrolide synthesis, cytotoxic marine natural product

## Abstract

β-Hydroxy aldehyde and alkyl ketone moieties were effectively synthesized as key intermediates of amphidinolide Q, a cytotoxic macrolide from the cultured dinoflagellate *Amphidinium* sp.. The asymmetric center of the former derivative was produced by Sharpless asymmetric epoxidation, followed by *E*-selective 1,4-addition to give the sp^2^ methyl group. Derivatization of the L-ascorbic acid derivative by Evans asymmetric alkylation and Peterson olefination provided the latter intermediate. The coupling reaction of the segments was examined.

## 1. Introduction 

**Scheme 1:**
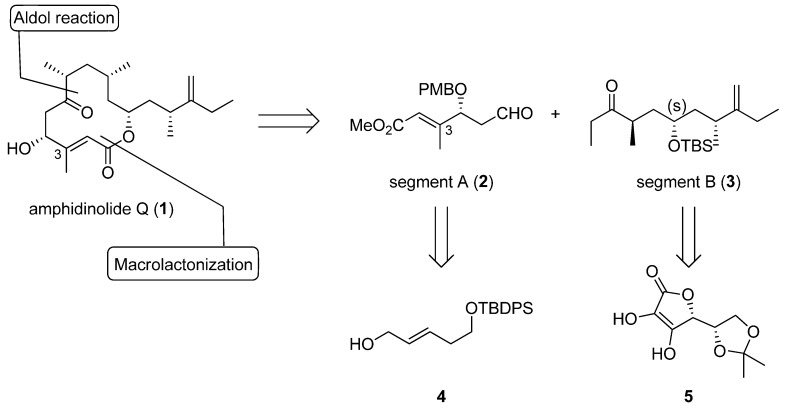
Amphidinolide Q (1) and retrosynthetic analysis.

Amphidinolide Q (**1**, Scheme 1) is a member of the cytotoxic macrolide family isolated from the cultured dinoflagellate *Amphidinium* sp., and its absolute stereostructure was chemically determined by Kobayashi and co-workers [1,2]. This macrolide exhibits potent cytotoxicity against L1210 murine leukemia cells. Due to its low natural abundance, the detailed structure-activity relationship of **1** has not been established. Although **1** was synthesized by Kobayashi’s group in 2009 [3], further increases in synthetic efficiency and examination of related substances based on new synthetic approaches are required to explain the detailed mode of action of **1** by structure-activity relationship studies, and to develop new bioactive substances superior to the mother macrolide. We describe herein an efficient method for synthesis of segments **2** and **3**, which will be important intermediates for the construction of **1**. 

## 2. Results and Discussion

As outlined in Scheme 1, retrosynthetic analysis showed that amphidinolide Q (**1**) could be obtained by successive coupling of **2** and **3**, with the former, carrying a stereogenic center and a tri-substituted olefin, being produced from the known allyl alcohol **4** through *E*-selective 1,4-addition of a methyl group at C3 and Sharpless asymmetric epoxidation. The alkyl ketone **3** carrying three stereogenic centers could be obtained from the ascorbic acid derivative **5** through Evans asymmetric alkylation. 

### 2.1. Synthesis of segment A *(**2**)*

**Scheme 2:**
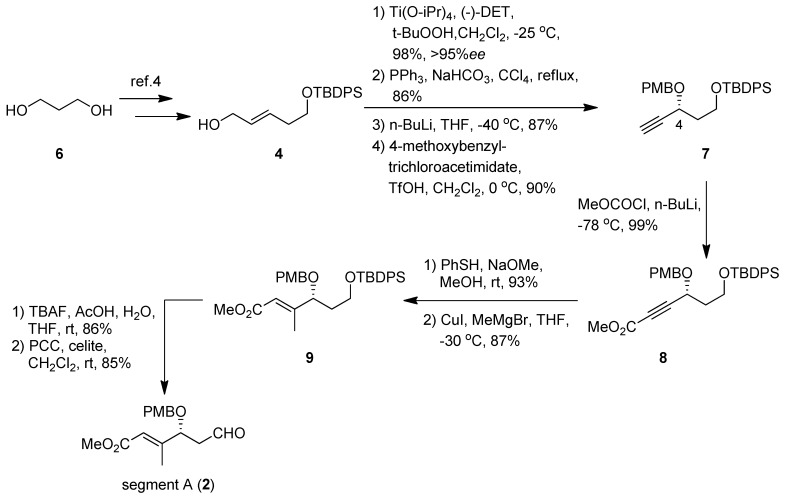
Synthesis of segment A (2).

The synthesis of **2** commenced with the conversion of 1,3-propanediol (**6**) into alcohol **4** (Scheme 2) [4]. Incorporation of the asymmetric center at C4 and carbon chain elongation were carried out by Sharpless asymmetric epoxidation, followed by chlorination, alkynylation [5], and final *p*-methoxy-benzyl (PMB) protection of the newly produced OH group under acidic conditions to avoid unexpected removal of the *tert*-butyldiphenylsilyl (TBDPS) group. Further manipulation of **7** involved introduction of a methoxycarbonyl group at the terminal of the alkynyl carbon to give **8**, which upon *E*-selective alkylation using a PhS group as an auxiliary [6], afforded the α,β-unsaturated ester **9**. In this case, the two-step procedure involving preparation of the vinyl sulfide, followed by exchange with a methyl group must be used to afford the α,β-unsaturated ester **9** in good yield (Table 1). In the case of direct methylation (path A), the desired compound **9** was obtained in low yield, even when the reaction temperature was increased to 0 °C (entry 2). Although a number of reagent combinations to control the properties of cuprates are known, the desired product was obtained in good yield in the case of entry 5. 

Deprotection and oxidation processes ultimately gave the β-hydroxyl aldehyde **2** (segment A) in good overall yield.

### 2.2. Synthesis of segment B *(**3**)*

**Scheme 3:**
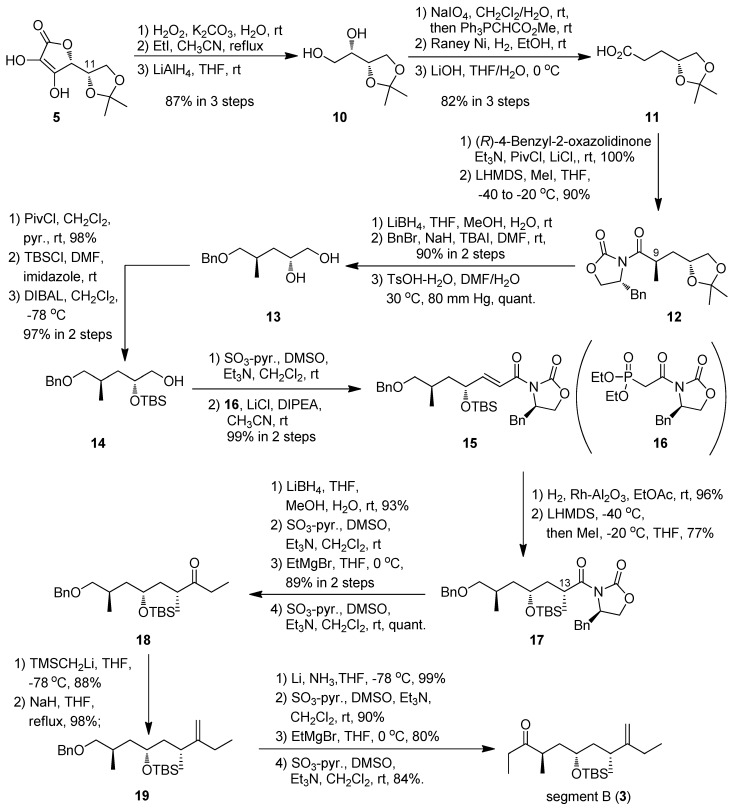
Synthesis of segment B (3).

Synthesis of alkyl ketone **3** was initiated by transformation of the ascorbic acid derivative **5** into diol **10** by a known procedure [7] (Scheme 3). 

Compound **10** was subjected to oxidative cleavage of the vicinal diol, followed by immediate Wittig reaction, hydrogenation, and hydrolysis to give **11**. In particular, the second Wittig reaction was carried out using crude aldehyde to avoid undesired epimerization of the asymmetric center at C11. Selective asymmetric induction at C9 by the Evans protocol effected the desired introduction of a methyl group to yield **12**. Removal of the chiral auxiliary by LiBH_4_ and manipulation of protecting groups in five steps gave the alcohol **14** via **13**. After Parikh–Doering oxidation of **14**, the aldehyde generated was reacted with Horner–Wadsworth–Emmons reagent **16** [8] to yield **15**, which on hydrogenation followed by addition of a methyl group produced **17**. After removal of the auxiliary by LiBH_4_, an ethyl ketone function was constructed (compound **18**), and the *exo*-methylene function was introduced by Peterson olefination, followed by basic treatment, whereas other methods, such as the Wittig and Petasis reactions did not produce the desired compound, probably due to the low reactivity of the ethylketone moiety. Synthesis of segment B (**3**) was accomplished by Birch reduction, followed by alkylation similar to that described the case of **18** and Parikh–Doering oxidation and Grignard reaction. 

The synthetic strategies used for both segments may enable the synthesis of analogs. The stereogenic centers were introduced via asymmetric reactions, which can produce another enantiomer by exchanging the auxiliary group or catalyst, with the exception of the C11 center: in this case the opposite (*R*)-enantiomer would be produced using (*R*)-glyceraldehyde acetonide, derived from D-mannitol [9].

**Scheme 4:**
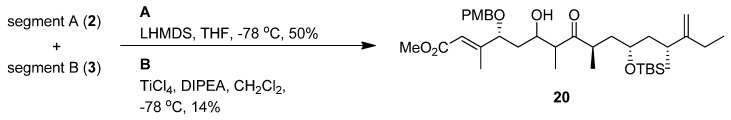
Coupling attempts between 2 and 3 [12].

Aldol coupling of both segments was attempted, as shown in Scheme 4. Whereas Lewis acidic conditions such as method B [10] caused undesirable elimination of **2** and **20** [11] to give the corresponding α,β,δ,γ-unsaturated ester, basic conditions (method A) provided **20**, equipped with the complete carbon framework, in moderate yield,. Detailed studies of the diastereomer distribution to improve the coupling yields are currently in progress in our laboratory.

## 3. Experimental 

### General

All reactions were carried out under an argon atmosphere unless otherwise noted. When necessary, solvents were dried prior to use. Dry THF, dry Et_2_O and dry CH_2_Cl_2_ were purchased from Kanto Chemical Co., Inc. Optical rotations were measured on a JASCO P-2200 digital polarimeter with a sodium (D line) lamp. IR spectra were recorded on a Jasco Model A-202 spectrophotometer. ^1^H-NMR spectra and ^13^C-NMR spectra were obtained on JEOL JNM-EX270, JNM-GX400, JNM-α400, JNM-AL400 and JNM-ECX400 spectrometers in deuterated solvent using tetramethylsilane as an internal standard. Deuteriochloroform was used as a solvent, unless otherwise stated. Optical purity was determined by HPLC using an OJ-H column. High-resolution mass spectra were obtained on a Waters LCT Piemier XE (ESI) or JEOL JMS-700 (FAB). Preparative and analytical TLC were carried out on silica gel plate (Kieselgel 60 F254, E. Merck AG., Germany) using UV light and/or 5% molybdophosphoric acid in ethanol for detection. Kanto silica 60N (spherical, neutral, 105–210 μm) was used for column chromatography.

*(R)-tert-Butyl(3-(4-methoxybenzyloxy)pent-4-ynyloxy)diphenylsilane* (**7**). To a suspension of Ti(OiPr)_4_ (4.28 mL, 14.6 mmol) and MS4A (4.9 g) in CH_2_Cl_2_ (100 mL) was added (-)-DET (3.32 mL, 19.5 mmol) at −25 °C. To the mixture were added **4** (3.31 g, 9.73 mmol) and t-BuOOH (3.17 mL, 29.2 mmol); the solution was stirred overnight. The reaction was quenched by L-(+)-tartaric acid (12.4 g, 82.6 mmol) aqueous and iron (II) sulfate heptahydrate (12.2 g, 43.9 mmol). The mixture was extracted with Et_2_O, and the organic extracts were dried (Na_2_SO_4_), and concentrated *in vacuo*. The crude product was purified by silica gel column chromatography using hexane-ethyl acetate (3:1) to give an alcohol (3.38 g, 98%, 95% *ee*) as a colorless oil: [α]^23^_D_ +14.7 (c 1.00, CHCl_3_); IR (film) 3437, 3048, 1428, 1110, 936, 822 cm^−1^; ^1^H-NMR (400 MHz) δ 1.06 (9H, s), 1.81 (2H, td, *J* = 12.1, 6.0 Hz), 2.98 (1H, td, *J* = 4.7. 2.5 Hz), 3.13 (1H, td, *J* = 6.0, 2.5 Hz), 3.63 (1H, m), 3.80 (2H, m), 3.91 (1H, m), 7.40 (6H, m), 7.66 (4H, m); ^13^C-NMR (100 MHz) δ 19.2, 26.8, 34.8, 53.7, 58.5, 60.7, 61.6, 127.7, 129.7, 133.6, 135.5. ESI-MS: calcd for C_21_H_29_O_3_Si 357.1886 (M+H)^+^, found, *m/z* 357.1897.

A stirred mixture of the alcohol (3.38 g, 9.49 mmol), PPh_3_ (7.47 g, 28.5 mmol), and NaHCO_3_ (0.80 g, 9.5 mmol) in CCl_4_ (100 mL) was refluxed under argon atmosphere overnight. After completion of the reaction, CCl_4_ was removed under reduced pressure, and the residue was purified by silica gel column chromatography (hexane-ethyl acetate 40:1) to furnish an epoxy chloride (3.04 g, 86%) as a colorless oil: [α]^23^_D_ +12.9 (c 1.00, CHCl_3_); IR (film) 3049, 1428, 1111, 939, 822 cm^−1^; ^1^H-NMR (400 MHz) δ 1.06 (9H, s), 1.81 (2H, td, *J* = 11.2, 5.6 Hz), 3.07 (2H, m), 3.56 (2H, m), 3.80 (2H, m), 7.41 (6H, m), 7.66 (4H, m); ^13^C-NMR (100 MHz) δ 19.2, 26.8, 34.7, 44.7, 56.7, 57.3, 60.5, 127.7, 129.7, 133.5, 135.5. ESI-MS: calcd for C_21_H_28_O_2_SiCl 375.1547 (M+H)^+^, found, *m/z* 375.1539.

To a stirred solution of the epoxy chloride (0.23 g, 0.63 mmol) in dry THF (7 mL) was added n-BuLi (2.4 mL, 1.6 M solution in *n*-hexane, 3.79 mmol) dropwise at −40 °C under argon atmosphere; and the mixture was stirred for an additional 2 h. The mixture was quenched with saturated aqueous NH_4_Cl, and the mixture was extracted with ethyl acetate. The organic extracts were dried (Na_2_SO_4_), and concentrated *in vacuo*. The residue was purified by silica gel column chromatography (hexane-ethyl acetate 7:1) to give an alcohol (0.18 g, 87%) as a colorless oil: [α]^23^_D_ +3.67 (c 1.00, CHCl_3_); IR (film) 3422, 3304, 3071, 1428, 1111, 823 cm^−1^; ^1^H-NMR (400 MHz) δ 1.06 (9H, s), 1.92 (1H, tdd, *J* = 14.0, 6.3, 3.6 Hz), 2.05 (1H, tdd, *J* = 14.0, 8.0, 4.3 Hz), 2.48 (1H, d, *J* = 5.8 Hz), 3.34 (1H, d, *J* = 5.8 Hz), 3.85 (1H, ddd, *J* = 12.0, 6.3, 4.3 Hz), 4.07 (1H, ddd, *J* = 12.0, 8.0, 3.6 Hz), 4.71 (1H, m), 7.42 (6H, m), 7.69 (4H, m); ^13^C-NMR (100 MHz) δ 19.0, 26.8, 38.5, 61.5, 61.7, 73.0, 84.4, 127.8, 129.9, 132.8, 135.6. ESI-MS: calcd for C_21_H_27_O_2_Si 339.1780 (M+H)^+^, found, *m/z* 339.1775.

To a solution of the alcohol (0.10 g, 0.30 mmol) in anhydrous CH_2_Cl_2_ (7 mL) were added 4-methoxybenzyl trichloroacetimidate (0.493 g, 1.74 mmol) and TfOH (cat.) at 0 ºC, and the mixture was stirred overnight. The reaction was quenched with saturated aqueous NH_4_Cl, and extracted with ethyl acetate. The organic extracts were dried (Na_2_SO_4_), and concentrated *in vacuo*. The residue was purified by silica gel column chromatography (hexane-ethyl acetate 7:1) to give **7** (0.12 g, 90%) as a colorless oil: [α]^23^_D_ +45.1 (c 1.02, CHCl_3_); IR (film) 3286, 1514, 1249, 1111, 823 cm^−1^; ^1^H-NMR (400 MHz) δ 0.94 (9H, s), 1.91 (2H, m), 2.36 (1H, d, *J* = 2.1 Hz), 3.72 (5H, m), 4.34 (2H, m), 4.66 (1H, d, *J* = 11.2 Hz), 6.77 (2H, m), 7.20 (2H, m), 7.31 (6H, m), 7.56 (4H, m); ^13^C-NMR (100 MHz) δ 19.2, 26.8, 38.7, 55.2, 59.7, 65.0, 70.3, 73.8, 83.0, 113.8, 127.6, 129.6, 133.7, 135.5, 159.2. ESI-MS: calcd for C_29_H_34_O_3_SiK 497.1914 (M+K)^+^, found, *m/z* 497.1911.

*(R)-Metyl-6-(tert-Butyldiphenylsilyloxy)-4-(4-methoxybenzyloxy)hex-2-ynoate* (**8**). To a solution of **7** (50.8 mg, 0.11 mmol) in THF (1.5 mL) was added dropwise *n*-BuLi (0.3 mL, 1.6 M solution in hexane, 0.48 mmol) at −78 °C, and the mixture was stirred at the same temperature for 1 h. Methyl chloroformate (0.09 mL, 1.11 mmol) was added dropwise to the mixture; the resulting mixture was stirred at 0 °C for 2.5 h. After being quenched with saturated aqueous NH_4_Cl, the mixture extracted with ethyl acetate. The organic layer was dried (Na_2_SO_4_), and concentrated *in vacuo*. The residue was purified by silica gel column chromatography (hexane-ethyl acetate 9:1) to give **8** (56.4 mg, 99%) as a colorless oil: [α]^23^_D_ +59.8 (*c* 0.99, CHCl_3_); IR (film) 3071, 2233, 1719, 1249, 1111 cm^−1^; ^1^H-NMR (400 MHz) *δ* 1.00 (9H, s), 2.01 (2H, ddd, *J* = 12.7, 7.6, 6.0 Hz), 3.79 (8H, m), 4.43 (1H, d, *J* = 11.2 Hz), 4.55 (1H, d, *J* = 7.6, 6.0 Hz), 4.74 (1H, d, *J* = 11.2 Hz), 6.86 (2H, m), 7.26 (2H, m), 7.37 (6H, m), 7.60 (4H, m); ^13^C-NMR (100 MHz) *δ* 19.1, 26.7, 38.0, 52.8, 55.2, 59.3, 64.7, 71.0, 86.9, 113.8, 127.7, 129.2, 129.6, 129.7, 133.5, 135.5, 153.7, 159.4. ESI-MS: calcd for C_31_H_36_O_5_SiK 555.1969 (M+K)^+^, found, *m/z* 555.1970.

*(R,E)-Methyl 6-(tert-butyldiphenylsilyloxy)-4-(4-methoxybenzyloxy)-3-methylhex-2-enoate* (**9**). To a solution of **8** (90.8 mg, 0.18 mmol) in MeOH was added PhSH (36 μL, 0.35 mmol) and NaOMe (0.18 mL, 0.02 mmol) at room temperature. After being stirred at the same temperature for 3 h, the reaction mixture was concentrated *in vacuo*. The residue was purified by silica gel column chromatography (hexane- ethyl acetate 9:1) to give a methyl ester (102.1 mg, 93%) as a colorless oil: [α]^23^_D_ -91.3 (*c* 1.0, CHCl_3_); IR (film) 1705, 1513, 1248, 1110 cm^−1^; ^1^H-NMR (400 MHz) *δ* 0.93 (9H, s), 1.63 (1H, m), 2.02 (1H, m), 3.46 (1H, m), 3.50 (1H, m), 3.77 (3H, s), 3.79 (3H, s), 3.96 (1H, dd, *J* = 2.5, 9.9 Hz), 4.04 (1H, d, *J* = 10.8 Hz), 4.46 (1H, d, *J* = 10.8 Hz), 6.35 (1H, s), 6.79 (2H, m), 7.07 (2H, m), 7.21 (3H, m), 7.34 (4H, m), 7.43 (4H, m), 7.53 (4H, m); ^13^C-NMR (100 MHz) *δ* 19.1, 26.8, 39.8, 51.4, 55.3, 60.2, 70.7, 75.3, 113.8, 111.6, 113.7, 127.5, 129.2, 129.3, 129.5, 135.3, 135.5, 159.2, 160.5, 166.8. ESI-MS: calcd for C_37_H_42_O_5_SiSNa 649.2420 (M+Na)^+^, found, *m/z* 649.2440.

To a suspension of CuI (0.62 g, 3.2 mmol) in THF was added MeMgBr (6.7 mL, 0.95 M in THF, 6.3 mmol) at −78 °C; the solution was warmed to −30 °C and stirred for 1.5 h. The solution was cooled to −78 °C, and the methyl ester (0.10 g, 0.16 mmol) was added. The reaction mixture was stirred at −30 °C for 1 h, then quenched with saturated aqueous NH_4_Cl and NH_4_OH. The reaction mixture was extracted with ethyl acetate, and the organic layer was dried (Na_2_SO_4_) and concentrated *in vacuo*. The residue was purified by silica gel column chromatography (benzene-hexane 7:1) to give **9** (75 mg, 87%) as a colorless oil: [α]^23^_D_ +64.0 (*c* 0.50, CHCl_3_); IR (film) 1720, 1428, 1249, 1111, 822 cm^−1^; ^1^H-NMR (400 MHz) *δ* 0.96 (9H, s), 1.69 (2H, m), 2.05 (3H, d, *J* = 1.0 Hz), 3.64 (8H, m), 3.99 (1H, t, *J* = 6.6 Hz), 4.08 (1H, d, *J* = 11.2 Hz), 4.34 (1H, d, *J* = 11.2 Hz), 5.86 (1H, s), 6.77 (2H, m), 7.12 (2H, m), 7.31 (6H, m), 7.56 (4H, m); ^13^C-NMR (100 MHz) *δ* 14.3, 19.2, 26.8, 37.2, 51.0, 55.2, 59.9, 70.6, 80.2, 113.8, 116.4, 127.6, 129.4, 129.6, 130.1, 133.7, 135.5, 158.9, 159.2, 167.0. ESI-MS: calcd for C_32_H_40_O_5_SiNa 555.2543 (M+Na)^+^, found, *m/z* 555.2530.

*(R,E)-Methyl 4-(4-methoxybenzyloxy)-3-methyl-6-oxohex-2-enoate* (**segment A**, **2**). To a solution of **9** (0.33 g, 0.62 mmol) in THF (6 mL) was added reagent (TBAF-AcOH-H_2_O= 1:1:5 0.1 M in THF, 0.3 mmol) at room temperature; the mixture was stirred at the same temperature overnight. After the addition of cold water, the mixture was extracted with Et_2_O. The organic layer was dried (Na_2_SO_4_) and concentrated *in vacuo*. The residue was purified by silica gel column chromatography (hexane-ethyl acetate 1:1) to give an alcohol (0.16 g, 88%) as a colorless oil: [α]^23^_D_ +68.7 (*c* 0.99, CHCl_3_); IR (film) 3429, 1718, 1654, 1613, 1513, 1156, 1035 cm^−1^; ^1^H-NMR (270 MHz) *δ* 1.84 (2H, m), 2.15 (3H, d, *J* = 1.3 Hz), 3.73 (5H, m), 3.81 (3H, s), 3.98 (1H, dd, *J* = 9.1, 3.8 Hz), 4.20 (1H, d, *J* = 11.2 Hz), 4.48 (1H, d, *J* = 11.2 Hz), 5.95 (1H, s), 6.89 (2H, m), 7.23 (2H, m); ^13^C-NMR (67.5 MHz) *δ* 14.4, 29.7, 36.3, 51.1, 55.2, 60.4, 70.6, 82.03, 113.9, 116.6, 128.3, 129.5, 129.6, 157.7, 159.4, 166.8. ESI-MS: calcd for C_16_H_22_O_5_Na 317.1365 (M+Na)^+^, found, *m/z* 317.1363.

To a mixture of PCC (35 mg, 0.16 mmol) and Celite in CH_2_Cl_2_ (2 mL) was added the alcohol (31.7 mg, 0.11 mmol) at 0 °C; the mixture was stirred at room temperature for 30 min. After concentration of the reaction mixture, the residue was purified by silica gel column chromatography (Et_2_O-hexane 2:1) to give **segment A** (26.7 mg, 85%) as a colorless oil: [α]^23^_D_ +28.9 (*c* 0.91, CHCl_3_); IR (film) 1720, 1655, 1612, 1513, 1248, 1034 cm^−1^; ^1^H-NMR (270 MHz, C_6_D_6_) *δ* 1.90 (1H, ddd, *J* = 1.1, 3.6, 16.6 Hz), 2.14 (3H, m), 2.34 (1H, ddd, *J* = 2.5, 9.2, 16.6 Hz), 3.38 (3H, s), 3.53 (3H, s), 4.05 (1H, m), 4.07 (1H, d, *J* = 11.2 Hz), 4.32 (1H, d, *J* = 11.2 Hz), 6.09 (1H, m), 6.84 (2H, m), 7.18 (2H, m), 9.38 (1H, dd, *J* = 1.1, 2.5 Hz); ^13^C-NMR (67.5 MHz, C_6_D_6_) *δ* 14.3, 47.5, 50.7, 54.7, 70.7, 77.6, 78.2, 114.1, 117.4, 129.7, 130.0, 156.6, 166.3, 198.3. ESI-MS: calcd for 293.1389 (M+H)^+^, found, *m/z* 293.1402.

*(R)-3-(2,2-Dimethyl-1,3-dioxolan-4-yl)propanoic acid* (**11**). To a solution of NaIO_4_ (3.78 g, 17.7 mmol) in CH_2_Cl_2_ (60 mL) and H_2_O (30 mL) was added **10** (1.91 g, 11.8 mmol) at 0 °C; the mixture was stirred at room temperature for 1.5 h. After the addition of Ph_3_PCHCO_2_Me (7.89 g, 23.6 mmol) at 0 °C, the mixture was stirred at room temperature overnight. The organic layer was separated and the aqueous layer was extracted with CHCl_3_ three times. The combined organic layers were washed with brine, dried (Na_2_SO_4_), and concentrated *in vacuo*. The residue was purified by silica gel column chromatography (hexane-ethyl acetate 3:1) to give an α,β-unsaturated ester (1.84 g, 84%, *E*:*Z* = 1:3) as a colorless oil. 

The ester (1.02 g, 5.48 mmol) was dissolved in EtOH (55 mL) in the presence of Raney Ni W-4. The mixture was stirred at room temperature under hydrogen atmosphere overnight. After filtration, the filtrate was concentrated at 110 °C to afford the corresponding ester as a crude oil. To a solution of the ester in THF (17 mL) was added 1.5 M LiOH aqueous (17 mL) at 0 °C. The mixture was stirred at 0 °C for 3 h. The mixture was acidified to pH 4 with 10% aqueous citric acid and extracted with ethyl acetate three times. The combined organic layers were washed with brine, dried (Na_2_SO_4_), and concentrated *in vacuo* to give **11** (0.935 g, 98%) as a colorless oil: [α]^23^_D_ +1.2 (*c* 1.00, CHCl_3_); IR (film) 2987, 1712, 1215, 1154, 1073 cm^−1^; ^1^H-NMR (270 MHz) *δ* 1.35 (3H, s), 1.42 (3H, s), 1.89 (2H, m), 2.51 (2H, m), 3.57 (1H, dd, *J* = 6.5, 7.8 Hz), 4.06 (1H, dd, *J* = 5.9, 7.8 Hz), 4.14 (1H, m); ^13^C- NMR (67.5 MHz) *δ* 25.6, 26.9, 28.5, 30.2, 68.9, 74.7, 109.1, 178.9. ESI-MS: calcd for C_8_H_15_O_4_ 175.0970 (M+H)^+^, found, *m/z* 175.0968.

*(R)-4-Benzyl-3-((R)-3-((R)-2,2-dimethyl-1,3-dioxolan-4-yl)-2-methylpropanoyl)oxazolidin-2-one* (**12**). To a solution of **11** (6.10 g, 35.0 mmol) in THF (325 mL) were added Et_3_N (15.0 mL, 0.190 mol) and PivCl (6.40 mL, 52.5 mmol) at 0 °C. After 2 h, LiCl (7.42 g, 175 mmol) and (*R*)-4-benzyl-2-oxazolidinone (9.30 g, 52.5 mmol) were added to the reaction mixture, and then the mixture was stirred at room temperature overnight. The reaction was quenched by the addition of saturated aqueous NH_4_Cl at 0 °C, then the resulting slurry was extracted with ethyl acetate three times. The combined organic layers were washed with brine, dried (Na_2_SO_4_), and concentrated *in vacuo*. The residue was purified by silica gel column chromatography (hexane-ethyl acetate 3:1) to give an amide (10.8 g, 92%) as a colorless oil: [α]^24^_D_ -42.4 (*c* 1.01, CHCl_3_); IR (film) 2984, 1782, 1690, 1382, 1212, 1054 cm^−1^; ^1^H-NMR (400 MHz) *δ* 1.35 (3H, s), 1.42 (3H, s), 1.96 (2H, m), 2.78 (1H, dd, *J* = 9.8, 13.2 Hz), 3.07 (2H, t, *J* = 7.8 Hz), 3.30 (1H, dd, *J* = 3.4, 13.2 Hz), 3.60 (1H, dd, *J* = 6.8, 7.8 Hz), 4.08 (1H, dd, *J* = 5.9, 7.8 Hz), 4.64 (3H, m), 4.67 (1H, m), 7.27 (5H, m); ^13^C-NMR (100 MHz) *δ* 25.7, 27.0, 28.2, 32.0, 37.9, 55.1, 66.2, 69.2, 74.9, 108.9, 127.2, 128.8, 129.3, 135.1, 153.3, 172.5, 180.1. ESI-MS: calcd for C_18_H_23_NO_5_Na 356.1474 (M+Na)^+^, found, *m/z* 356.1489.

To a solution of LHMDS (73.0 mL, 1.0 M solution in THF, 73.0 mmol) was added the amide (12.2 g, 36.5 mmol) in THF (400 mL) at −40 °C. After 1.5 h, MeI (22.7 mL, 0.365 mol) was added to the reaction mixture. The mixture was stirred at −20 °C for 4 h. The reaction was quenched by the addition of saturated aqueous NH_4_Cl at 0 °C, then the resulting slurry was extracted with ethyl acetate three times. The combined organic layers were washed with brine, dried (Na_2_SO_4_), and concentrated *in vacuo*. The residue was purified by silica gel column chromatography (hexane-ethyl acetate 3:1) to give **12** (10.8 g, 85%) as a white needles: m.p. 93–93.5 °C (ethyl acetate); [α]^24^_D_ -65.4 (*c* 1.00, CHCl_3_); IR (film) 2983, 1780, 1697, 1381, 1213, 1159, 1085, 1053 cm^−1^; ^1^H-NMR (400 MHz) *δ* 1.26 (3H, d, *J* = 7.3 Hz), 1.30 (3H, s), 1.36 (3H, s), 1.66 (1H, ddd, *J* = 4.4, 5.4, 13.7 Hz), 2.05 (1H, ddd, *J* = 8.8, 8.3, 13.7 Hz), 2.79 (1H, dd, *J* = 9.8, 13.2 Hz), 3.26 (1H, dd, *J* = 3.4, 13.2 Hz), 3.50 (1H, dd, *J* = 7.3, 7.8 Hz), 4.01 (1H, m), 4.03 (1H, dd, *J* = 5.4, 7.8 Hz), 4.17 (3H, m), 4.67 (1H, m), 7.28 (5H, m); ^13^C- NMR (100 MHz) *δ* 18.2, 25.7, 26.9, 34.7, 38.0, 38.1, 55.4, 66.0, 69.5, 74.2, 108.9, 127.2, 128.8, 129.3, 153.2, 176.9, 180.1. ESI-MS: calcd for C_19_H_26_NO_5_Na (M+Na)^+^ 348.1811, found, *m/z* 348.1805.

*(2R,4R)-5-(Benzyloxy)-4-methylpentane-1,2-diol* (**13**). To a solution of **12** (7.44 g, 21.4 mmol) in THF-MeOH-H_2_O (4:4:1), (220 mL) was added LiBH_4_ (2.1 g, 86 mmol) at 0 °C. The mixture was stirred at room temperature for 2.5 h. The reaction was quenched by the addition of saturated aqueous NH_4_Cl at 0 °C, then the resulting slurry was extracted with Et_2_O three times. The combined organic layers were washed with brine, dried (Na_2_SO_4_), and concentrated *in vacuo*. The residue was purified by silica gel chromatography (hexane-ethyl acetate (1:1)) to give an alcohol. To a solution of the alcohol in DMF (220 mL) was added BnBr (8 mL, 64.3 mmol), TBAI (3.2 g, 8.57 mmol) and NaH (3.1 g, 64.3 mmol) at 0 °C. The mixture was stirred at room temperature overnight. The reaction was quenched by the addition of saturated aqueous NH_4_Cl at 0 °C, then the resulting slurry was extracted with Et_2_O three times. The combined organic layers were washed with brine, dried (Na_2_SO_4_), and concentrated *in vacuo*. The residue was purified by silica gel column chromatography (hexane-ethyl acetate 10:1) to give an acetonide (5.09 g, 90%) as a colorless oil: [α]^24^_D_ -11.5 (*c* 1.03, CHCl_3_); IR (film) 2984, 1454, 1368, 1213, 1098, 1061 cm^−1^; ^1^H-NMR (400 MHz) *δ* 0.91 (3H, d, *J* = 6.8 Hz), 1.29 (7H, m), 1.71 (1H, ddd, *J* = 5.8, 8.3, 13.7 Hz), 1.89 (1H, m), 3.24 (2H, m), 3.42 (1H, t, *J* = 7.8 Hz), 3.97 (1H, dd, *J* = 7.8, 5.8 Hz), 4.12 (1H, tt, *J* = 7.8, 5.8 Hz), 4.43 (2H, s), 7.26 (5H, m); ^13^C-NMR (100 MHz) *δ* 17.1, 25.8, 27.1, 30.7, 37.8, 69.9, 72.9, 74.2, 108.5, 127.4, 127.5, 128.3, 138.6. ESI-MS: calcd for C_16_H_25_O_3_ 265.1804 (M+H)^+^, found, *m/z* 265.1803.

To a solution of the acetonide (0.73 g, 2.8 mmol) in DMF (30 mL) were added TsOH·H_2_O (1.6 g, 8.3 mmol) and H_2_O (10 mL) at 0 °C; the mixture was stirred at 30 ºC under 80 mmHg for 3 h. After the addition of saturated aqueous NH_4_Cl, the mixture was extracted with Et_2_O. The organic layer was dried (Na_2_SO_4_), and concentrated *in vacuo*. The residue was purified by silica gel column chromatography (hexane-ethyl acetate 1:1) to give **13** (0.66 g, quant.) as a colorless oil: [α]^24^_D_ +16.0 (*c* 0.99, CHCl_3_); IR (film) 3375, 1454, 1363, 1074 cm^−1^; ^1^H-NMR (400 MHz) *δ* 0.87 (3H, d, *J* = 6.8 Hz), 1.39 (2H,m), 1.93 (2H, m), 3.22 (1H, m), 3.37 (2H, m), 3.52 (2H, m), 3.70 (1H, m), 4.47 (2H, s), 7.24 (5H, m); ^13^C- NMR (100 MHz) *δ* 18.2, 31.8, 39.6, 67.4, 71.0, 73.3, 76.6, 127.7, 127.8, 128.5, 137.6. ESI-MS: calcd for C_13_H_20_O_3_Na 247.1310 (M+Na)^+^, found, *m/z* 247.1309.

*(2R,4R)-5-(Benzyloxy)-2-(tert-butyldimethylsilyloxy)-4-methylpentan-1-ol* (**14**). To a solution of **13** (3.39 g, 15.1 mmol) in CH_2_Cl_2_ (75 mL) and pyridine (75 mL) was added PivCl (2.8 mL, 22.7 mmol) at 0 °C; the mixture was stirred at room temperature for 1.5 h. The reaction was quenched with saturated aqueous NH_4_Cl, and the mixture was extracted with Et_2_O. The organic layer was dried (Na_2_SO_4_), and concentrated *in vacuo*. The residue was purified by silica gel column chromatography (hexane-ethyl acetate 4:1) to give an alcohol (4.57 g, 98%) as a colorless oil: [α]^24^_D_ +10.7 (*c* 1.01, CHCl_3_); IR (film) 3442, 1730, 1455, 1285, 1164, 1097 cm^−1^; ^1^H-NMR (400 MHz) *δ* 0.88 (3H, d, *J* = 7.3 Hz), 1.15 (9H, s), 1.33 (1H, ddd, *J* = 14.4, 6.8, 2.9 Hz), 1.46 (1H, m), 1.95 (1H, m), 2.98 (1H, br), 3.22 (1H, dd, *J* = 9.8, 6.3 Hz), 3.32 (1H, dd, *J* = 9.8, 5.4 Hz), 3.84 (1H, m), 3.96 (2H, m), 4.46 (2H, s), 7.26 (5H, m); ^13^C-NMR (100 MHz) *δ* 17.7, 27.2, 31.2, 38.8, 39.0, 68.8, 73.1, 76.2, 127.7, 128.3, 128.4, 138.0, 178.6. ESI-MS: calcd for C_18_H_28_O_4_Na 331.1885 (M+Na)^+^, found, *m/z* 331.1880.

To a solution of the alcohol (4.57 g, 14.8 mmol) in DMF (150 mL) were added imidazole (3.03 g, 44.5 mmol) and TBSCl (6.7 g, 44.5 mmol) at 0 °C; the mixture was stirred at room temperature overnight. After the addition of saturated aqueous NH_4_Cl, the mixture was extracted with Et_2_O. The organic layer dried (Na_2_SO_4_), and concentrated *in vacuo*. The residue was purified by silica gel column chromatography (hexane-ethyl acetate 7:1) to give a silyl ether (6.31 g, quant.) as a colorless oil: [α]^24^_D_ +10.6 (*c* 1.02, CHCl_3_); IR (film) 1733, 1160, 836 cm^−1^; ^1^H-NMR (400 MHz) *δ* 0.09 (6H, s), 0.88 (9H, s), 0.94 (3H, m), 1.20 (9H, s), 1.28 (1H, m), 1.63 (1H, ddd, *J* = 4.5, 7.9, 13.7 Hz), 1.97 (1H, m), 3.27 (1H, dd, *J* = 6.1, 9.2 Hz), 3.33 (1H, dd, *J* = 6.1, 9.2 Hz), 3.97 (3H, m), 4.50 (2H, s), 7.34 (5H, m); ^13^C- NMR (100 MHz) *δ* –4.7, –4.4, 17.1, 18.0, 25.5, 25.8, 27.1, 27.2, 29.5, 38.7, 38.8, 68.1, 68.4, 76.2, 127.3, 127.4, 128.3, 138.7, 178.5. ESI-MS: calcd for C_24_H_42_O_4_SiNa 445.2750 (M+Na)^+^, found, *m/z* 445.2747.

To a solution of the silyl ether (11.7 mg, 27.7 μmol) in CH_2_Cl_2_ (0.3 mL) was added DIBAL-H (0.08 mL, 1.03 M in n-hexane, 83.1 μmol) at −78 °C. After being stirred for 1 h then the reaction was quenched by potassium-sodium tartrate aqueous. The mixture was extracted with CHCl_3_, dried (Na_2_SO_4_), and concentrated *in vacuo*. The crude product was purified by silica gel column chromatography using hexane-ethyl acetate (5:1) to give **14** (9.1 mg, 97%) as a colorless oil: [α]^24^_D_ -2.3 (*c* 0.97, CHCl_3_); IR (film) 3437, 2857, 836 cm^−1^; ^1^H-NMR (400 MHz) *δ* 0.08 (6H, s), 0.90 (9H, s), 0.95 (3H, m), 1.29 (2H, m), 1.66 (1H, m), 1.90 (1H, m), 3.29 (2H, m), 3.45 (1H, dd, *J* = 4.9, 11.0 Hz), 3.58 (1H, dd, *J* = 4.9, 11.0 Hz), 3.88 (1H, m), 4.49 (2H, s), 7.33 (5H, m); ^13^C-NMR (100 MHz) *δ* –4.5, –4.4, 17.7, 18.1, 25.8, 29.6, 38.2, 66.6, 70.8, 73.0, 76.1, 127.4, 127.5, 128.3, 138.5. ESI-MS: calcd for C_19_H_34_O_3_SiNa 361.2175 (M+Na)^+^, found, *m/z* 361.2168.

*(R)-4-Benzyl-3-((4R,6R,E)-7-(benzyloxy)-6-methyl-4-(tert-butyldimethylsilyloxy)hept-2-enoyl)oxazol-idin-2-one* (**15**). To a solution of **14** (4.39 g, 13 mmol) in CH_2_Cl_2_ (65 mL) and DMSO (65 mL) were added Et_3_N (5.4 mL, 39 mmol) and SO_3_-pyridine (6.2 g, 39 mmol) complex at 0 °C. After 2 h, the reaction was quenched with saturated aqueous NH_4_Cl, and the mixture was partitioned between Et_2_O and H_2_O. The organic layer was dried (Na_2_SO_4_), and concentrated *in vacuo*. The residue was purified by silica gel column chromatography (hexane-ethyl acetate 6:1) to give an aldehyde (4.3 g, 99%) as a colorless oil. 

To a suspension of LiCl (0.09 g, 2.2 mmol) in anhydrous CH_3_CN (8 mL) were added **16** (0.29 g, 0.81 mmol), DIPEA (0.3 mL, 1.65 mmol), and the aldehyde (0.18 g, 0.55 mmol) at room temperature; the mixture was stirred at room temperature for overnight. The reaction was quenched with saturated aqueous NH_4_Cl, and the mixture was partitioned between Et_2_O and H_2_O. The organic layer was dried (Na_2_SO_4_), and concentrated *in vacuo*. The residue was purified by silica gel column chromatography (hexane-ethyl acetate 6:1) to give **15** (0.33 g, quant.) as a colorless oil: [α]^24^_D_ -23.1 (*c* 1.02, CHCl_3_); IR (film) 1782, 1683, 1639, 1352, 1207, 1100, 836 cm^−1^; ^1^H-NMR (400 MHz) *δ* 0.07 (6H, s), 0.93 (9H, s), 0.99 (3H, d, *J* = 6.8 Hz), 1.35 (1H, ddd, *J* = 4.9, 8.8, 13.7 Hz), 1.75 (1H, ddd, *J* = 4.9, 8.8, 13.7 Hz), 2.02 (1H, m), 2.81 (1H, dd, *J* = 9.8, 13.7 Hz), 3.32 (3H, m), 4.19 (2H, m), 4.50 (3H, m), 4.73 (1H, ddd, *J* = 3.4, 6.8, 12.7 Hz), 7.37 (12H, m); ^13^C-NMR (100 MHz) *δ* –5.0, –4.3, 17.3, 18.1, 25.8, 29.7, 37.9, 41.6, 55.3, 66.1, 70.2, 72.9, 76.0, 77.3, 118.7, 127.3, 127.4, 127.5, 128.3, 128.9, 129.5, 135.3, 138.6, 153.2, 153.6, 165.0. ESI-MS: calcd for C_31_H_43_NO_5_SiNa 560.2808 (M+Na)^+^, found, *m/z* 560.2808.

*(R)-4-Benzyl-3-((2R,4R,6R)-7-(benzyloxy)-2,6-dimethyl-4-(tert-butyldimethylsilyloxy)heptanoyl)oxazol-idin-2-one* (**17**). To a solution of **15** (6.21 g, 11.6 mmol) in ethyl acetate (120 mL) was added Rh-Al_2_O_3_ (cat.) at room temperature under hydrogen atmosphere; the mixture was stirred for 2 h. The reaction mixture was filtered and concentrated *in vacuo*. The residue was purified by silica gel column chromatography (hexane-ethyl acetate 6:1) to give an amide (5.99 g, 96%) as a colorless oil: [α]^24^_D_ -23.6 (*c* 0.99, CHCl_3_); IR (film) 1784, 1701, 1210, 835 cm^−1^; ^1^H-NMR (400 MHz,) *δ* 0.07 (6H, d, *J* = 4.9 Hz), 0.90 (9H, s), 0.97 (3H, d, *J* = 6.5 Hz), 1.26 (1H, ddd, *J* = 5.4, 8.1, 13.7 Hz), 1.62 (1H, ddd, *J* = 5.4, 7.2, 13.7 Hz), 1.88 (3H, m), 2.76 (1H, dd, *J* = 9.6, 13.7 Hz), 2.92 (1H, ddd, *J* = 5.4, 9.6, 17.3 Hz), 3.05 (1H, ddd, *J* = 5.4, 9.6, 17.3 Hz), 3.31 (3H, m), 3.89 (1H, tt, *J* = 5.4, 11.7 Hz), 4.17 (2H, m), 4.50 (2H, s), 4.66 (1H, ddd, *J* = 3.4, 7.2, 13.0 Hz), 7.34 (10H, m); ^13^C-NMR (100 MHz) *δ* –4.5, –4.4, 17.6, 18.0, 25.9, 29.8, 31.5, 31.7, 37.9, 41.3, 55.1, 66.1, 69.2, 72.8, 76.1, 127.4, 127.5, 128.2, 128.3, 128.9, 129.4, 135.3, 153.4, 173.4. ESI-MS: calcd for C_31_H_45_NO_5_SiNa 562.2965 (M+Na)^+^, found, *m/z* 562.2962.

To a solution of LHMDS (47 mL, 1.0 M in THF, 47 mmol) in THF (100 mL) was added the amide (5.07 g, 9.4 mmol) at −40 °C. After being stirred at same temperature for 2 h, MeI (12 mL, 0.19 mol) was added and the resultant mixture stirred at −20 °C for 0.5 h. After the addition of saturated aqueous NH_4_Cl, the mixture was extracted with ethyl acetate. The organic layer was dried (Na_2_SO_4_), and concentrated *in vacuo*. The residue was purified by silica gel column chromatography (hexane-ethyl acetate 9:1) to give **17** (4.03 g, 77%) as a colorless oil: [α]^24^_D_ -41.5 (*c* 1.00, CHCl_3_); IR (film) 1783, 1697, 1455, 1386, 1209, 836 cm^−1^; ^1^H-NMR (400 MHz) *δ* 0.05 (6H, d, *J* = 14.6 Hz), 0.90 (9H, s), 1.02 (3H, d, *J* = 6.1 Hz), 1.32 (4H, m), 1.58 (2H, m), 1.98 (1H, ddd, *J* = 6.7, 13.2, 19.7 Hz), 2.11 (1H, m), 2.80 (1H, dd, *J* = 10.1, 13.2 Hz), 3.34 (3H, m), 3.84 (2H, m), 4.18 (2H, m), 4.55 (2H, s), 4.67 (1H, m), 7.33 (10H, m); ^13^C-NMR (100 MHz) *δ* –4.7, –4.3, 17.9, 18.0, 18.6, 25.8, 29.9, 34.3, 37.9, 40.7, 41.6, 55.3, 65.9, 68.7, 72.9, 76.0, 127.3, 127.5, 128.2, 128.9, 129.4, 135.3, 138.8, 152.8, 176.8. ESI-MS: calcd for C_32_H_48_NO_5_Si 554.3302 (M+H)^+^, found, *m/z* 554.3292.

*(4R,6R,8R)-9-(Benzyloxy)-4,8-dimethyl-6-(tert-butyldimethylsilyloxy)nonan-3-one* (**18**). To a solution of **17** (4.7 g, 8.49 mmol) in THF-MeOH-H_2_O (4:4:1, 90 mL) was added LiBH_4_ (0.82 g, 34 mmol) at 0 °C; the mixture was stirred at room temperature for 1 h. After the addition of saturated aqueous NH_4_Cl, the mixture was extracted with Et_2_O. The organic layer was dried (Na_2_SO_4_), and concentrated *in vacuo*. The residue was purified by silica gel column chromatography (hexane-ethyl acetate 5:1) to give an alcohol (3.02 g, 93%) as a yellow oil: [α]^24^_D_ +9.1 (*c* 1.00, CHCl_3_); IR (film) 1459, 1255, 1049, 835 cm^−1^; ^1^H-NMR (400 MHz) *δ* 0.08 (6H, d, *J* = 3.6 Hz), 0.87 (3H, d, *J* = 7.2 Hz), 0.90 (9H, s), 0.95 (3H, d, *J* = 7.2 Hz), 1.38 (1H, ddd, *J* = 6.3, 7.2, 13.7 Hz), 1.55 (4H, m), 1.86 (2H, m), 3.26 (2H, m), 3.33 (1H, m), 3.44 (1H, m), 3.98 (1H, tt, *J* = 4.5, 11.4 Hz), 4.49 (2H, s), 7.33 (5H, m); ^13^C-NMR (100 MHz) *δ* –4.6, –4.4, 17.8, 18.0, 18.3, 25.8, 30.0, 31.8, 40.6, 42.6, 69.3, 73.0, 76.2, 127.4, 127.5, 128.3, 138.6. ESI-MS: calcd for C_22_H_41_O_3_Si 381.2825 (M+H)^+^, found, *m/z* 381.2821.

To a solution of the alcohol (2.5 mg, 6.6 μmol) in CH_2_Cl_2_ (0.05 mL) and DMSO (0.05 mL) were added Et_3_N (3 μL, 19.7 μmol) and SO_3_-pyridine (3 mg, 19.7 μmol) complex at 0 °C. After 1.5 h, the reaction was quenched with saturated aqueous NH_4_Cl, and the mixture was partitioned between Et_2_O and H_2_O. The organic layer was dried (Na_2_SO_4_), and concentrated *in vacuo*. The residue was purified by PLC (hexane-ethyl acetate 3:1) to give an aldehyde as a colorless oil.

To a solution of the aldehyde in THF (0.1 mL) was added EtMgBr (0.02 mL, 1.0 M in THF, 19.7 μmol) at 0 °C; the mixture was stirred at the same temperature for 15 min. The reaction mixture was quenched with saturated aqueous NH_4_Cl, and extracted with CH_2_Cl_2_. The organic layer was dried (Na_2_SO_4_), and concentrated *in vacuo*. The residue was purified by PLC (hexane-ethyl acetate (5:1)) to give an alcohol as a colorless oil.

To a solution of the alcohol in CH_2_Cl_2_ (0.05 mL) and DMSO (0.05 mL) were added Et_3_N (3 μL, 19.7 μmol) and SO_3_-pyridine (3 mg, 19.7 μmol) complex at 0 °C. After 4 h, the reaction was quenched with saturated aqueous NH_4_Cl, and the mixture was partitioned between Et_2_O and H_2_O. The organic layer was dried (Na_2_SO_4_), and concentrated *in vacuo*. The residue was purified by PLC (hexane-ethyl acetate (5:1) to give **18** (2.5 mg 89% in 3 steps) as a colorless oil: [α]^24^_D_ +1.1 (*c* 0.98, CHCl_3_); IR (film) 1714, 1459, 1255, 1099, 835 cm^−1^; ^1^H-NMR (400 MHz) *δ* 0.02 (6H, d, *J* = 6.7 Hz), 0.87 (9H, s), 0.95 (3H, d, *J* = 7.2 Hz), 1.05 (6H, m), 1.22 (1H, m), 1.35 (1H, m), 1.57 (1H, m), 1.93 (1H, m), 2.46 (2H, m), 2.66 (qt, 1H, *J* = 7.2, 13.7 Hz), 3.26 (1H, dd, *J* = 6.1, 8.8 Hz), 3.33 (1H, dd, *J* = 6.1, 8.8 Hz), 3.75 (1H, tt, *J* = 5.8, 12.1 Hz), 4.50 (2H, s), 7.33 (5H, m); ^13^C-NMR (100 MHz) *δ* –4.34, –4.30, 7.8, 17.5, 17.7, 18.1, 25.9, 29.8, 34.2, 40.6, 41.4, 42.3, 68.8, 75.9, 127.4, 127.5, 128.3, 138.8, 214.7. ESI-MS: calcd for C_24_H_43_O_3_Si 407.2981 (M+H)^+^, found, *m/z* 407.2982.

*((2R,4S,6R)-1-(Benzyloxy)-2,6-dimethyl-7-methylenenonan-4-yloxy)(tert-butyl)dimethylsilane* (**19**). To a solution of ethyl ketone **18** (60.0 mg, 0.12 mmol) in THF (1 mL) was added (trimethylsilylmethyl) lithium (0.5 mL, 1.0 M in pentane, 0.5 mmol) at −78 °C; the mixture was stirred at the same temperature for 20 min. The reaction mixture was quenched with saturated aqueous NH_4_Cl, and extracted with ethyl acetate. The organic layer was dried (Na_2_SO_4_), and concentrated *in vacuo*. The residue was purified by PLC (hexane-ethyl acetate 9:1) to give an alcohol as a colorless oil.

To a solution of the alcohol in THF (2 mL) was added NaH (60% dispersion in mineral oil, 50 mg, 0.1 mmol) at room temperature; the mixture was stirred at reflux temperature overnight. The reaction was quenched with saturated aqueous NH_4_Cl, and the mixture was extracted with ethyl acetate. The organic layer was dried (Na_2_SO_4_) and concentrated *in vacuo*. The residue was purified by PLC (hexane-ethyl acetate 9:1) to give **19** as a colorless oil (48 mg, 86% in 2 steps): [α]^24^_D_ +17.8 (*c* 1.01, CHCl_3_); IR (film) 2958, 1642, 1460, 1254, 1097, 835 cm^−1^; ^1^H-NMR (400 MHz) *δ* 0.05 (6H, s), 0.89 (9H, s), 0.95 (3H, d, *J* = 6.5 Hz), 1.02 (6H, m), 1.24 (1H, m), 1.46 (2H, m), 1.61 (1H, m), 1.99 (3H, m), 2.19 (1H, m), 3.23 (1H, dd, *J* = 6.5, 9.1 Hz), 3.34 (1H, dd, *J* = 6.5, 9.1 Hz), 3.77 (1H, m), 4.50 (2H, s), 4.72 (2H, dd, *J* = 1.5, 11.9 Hz), 7.33 (5H, m); ^13^C-NMR (100 MHz) *δ* –4.2, –4.1, 12.4, 17.4, 18.1, 20.3, 26.0, 26.2, 29.7, 36.8, 41.3, 44.2, 69.0, 72.8, 76.3, 106.4, 127.4, 127.5, 128.3, 138.8, 156.3. ESI-MS: calcd for C_25_H_45_O_2_Si 405.3189 (M+H)^+^, found, *m/z* 405.3181.

*(4R,6S,8R)-6-(tert-Butyldimethylsilyloxy)-4,8-dimethyl-9-methyleneundecan-3-one* (**segment B**, **3**). To a solution of lithium (12.4 mg, 1.9 mmol) in liquid NH_3_ (10 mL) was added **19** (46.4 mg, 0.11 mmol) in dry THF (3 mL) at −78 °C. The reaction mixture was stirred for 15 min at the same temperature; and quenched with solid NH_4_Cl. The mixture was extracted with ethyl acetate, and the organic layer was dried (Na_2_SO_4_), and concentrated *in vacuo*. The residue was purified by silica gel column chromatography (hexane-ethyl acetate 5:1) to give an alcohol (35.6 mg, 99%) as a colorless oil: [α]^24^_D_ +36.4 (*c* 0.98, CHCl_3_); IR (film) 3346, 2959, 1643, 1461, 1254, 1046, 835 cm^−1^; ^1^H-NMR (400 MHz) *δ* 0.06 (6H, d, *J* = 1.6 Hz), 0.89 (9H, s), 0.93 (3H, d, *J* = 6.3 Hz), 1.02 (6H, m), 1.27 (1H, ddd, *J* = 3.7, 8.5, 13.9 Hz), 1.46 (2H, m), 1.59 (1H, m), 1.81 (1H, ttd, *J* = 2.2, 6.3, 13.0 Hz), 2.00 (2H, m), 2.16 (1H, qt, *J* = 6.3, 7.0 Hz), 3.45 (2H, dd, *J* = 4.5, 6.3 Hz), 3.76 (1H, m), 4.72 (2H, ddd, *J* = 1.6, 2.9, 11.9 Hz); ^13^C-NMR (100 MHz) *δ* –4.2, –4.0, 12.4, 17.0, 18.1, 20.1, 25.8, 25.9, 26.3, 32.3, 36.9, 40.8, 44.4, 68.8, 69.3, 106.4, 156.3. ESI-MS: calcd for C_18_H_39_O_2_Si 315.2719 (M+H)^+^, found, *m/z* 315.2717.

To a solution of the alcohol (0.35 g, 1.10 mmol) in CH_2_Cl_2_ (6 mL) and DMSO (6 mL) were added Et_3_N (0.5 mL, 3.29 mmol) and SO_3_-pyridine (0.52 g, 3.29 mmol) complex at 0 °C. After 1 h, the reaction was quenched with saturated aqueous NH_4_Cl, and the mixture was partitioned between Et_2_O and H_2_O. The organic layer was dried (Na_2_SO_4_), and concentrated *in vacuo*. The residue was purified by silica gel column chromatography (hexane-ethyl acetate 7:1) to give an aldehyde as a colorless oil.

To a solution of the aldehyde in THF (10 mL) was added EtMgBr (3 mL, 1.0 M in THF, 2.95 mmol) at 0 °C; the mixture was stirred at the same temperature for 30 min. The reaction was quenched with saturated aqueous NH_4_Cl, and the mixture was extracted with CH_2_Cl_2_. The organic layer was dried (Na_2_SO_4_), and concentrated *in vacuo*. The residue was purified by silica gel column chromatography (hexane-ethyl acetate (9:1)) to give an alcohol as a colorless oil.

To a solution of the alcohol in CH_2_Cl_2_ (4 mL) and DMSO (4 mL) were added Et_3_N (0.32 mL, 2.34 mmol) and SO_3_-pyridine (0.4 g, 2.34 mmol) complex at 0 °C. After 40 min, the reaction was quenched with saturated aqueous NH_4_Cl, and the mixture was partitioned between Et_2_O and H_2_O. The organic layer was dried (Na_2_SO_4_), and concentrated *in vacuo*. The residue was purified by silica gel column chromatography (hexane-ethyl acetate 11:1) to give **segment B** (0.22 g 60% in 3 steps) as a colorless oil.: [α]^24^_D_ +11.7 (*c* 0.99, CHCl_3_); IR (film) 2960, 1716, 1643, 1461, 1255, 1101, 835 cm^−1^; ^1^H-NMR (400 MHz) *δ* 0.06 (6H, d, *J* = 3.8 Hz), 0.89 (9H, s), 1.02 (12H, m), 1.38 (2H, m), 1.55 (1H, ddd, *J* = 6.7, 7.0, 13.5 Hz), 1.74 (1H, ddd, *J* = 5.2, 7.0, 13.5 Hz), 1.97 (2H, m), 2.16 (1H, dd, *J* = 7.0, 14.0 Hz), 2.47 (2H, q, *J* = 7.0 Hz), 2.73 (1H, qt, *J* = 7.0, 14.0 Hz), 3.66 (1H, m), 4.70 (2H, m); ^13^C- NMR (100 MHz) *δ* –4.3, –4.1, 7.9, 12.3, 16.4, 18.1, 20.3, 25.9, 26.2, 34.2, 36.8, 40.1, 41.8, 44.0, 68.7, 77.3, 106.6, 156.0, 215.2. ESI-MS: calcd for C_20_H_39_O_2_Si 339.2719 (M+H)^+^, found, *m/z* 339.2689.

## 4. Conclusions

In conclusion, this study reports the efficient synthesis of promising segments **2** and **3** for the construction of amphidinolide Q (**1**). Both routes provided good yields and availability for analog synthesis.

## Figures and Tables

**Table 1:**
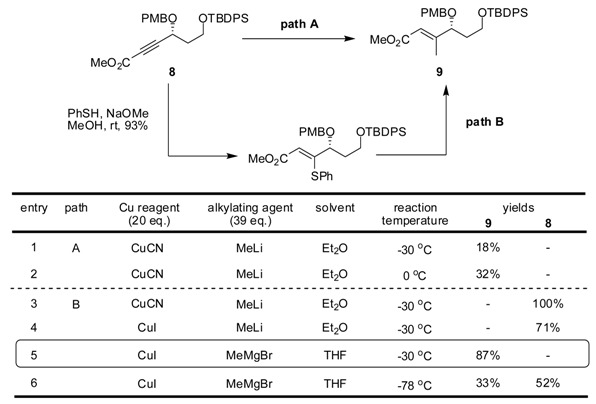
1,4-Addition of methyl group at C3 position of compound 6.
